# Recent Advances in the Management of IgA Nephropathy: From Supportive Care to Targeted Therapies

**DOI:** 10.7759/cureus.111227

**Published:** 2026-06-21

**Authors:** Abdulaziz Alroshodi

**Affiliations:** 1 Department of Medicine, College of Medicine, Qassim University, Qassim, SAU

**Keywords:** corticosteroids, iga nephropathy, sglt2 inhibitors, supportive therapy, targeted therapies

## Abstract

Immunoglobulin A nephropathy (IgAN) represents the predominant glomerulonephritis globally and is a leading cause of chronic kidney disease (CKD) and end-stage renal disease (ESRD). Historically, supportive management, with blood pressure control, renal angiotensin system (RAS) blockade, and active management with steroids, has been tried. However, the treatment landscape has recently become more complex with the introduction of several highly specific therapies. This review aims to provide an in-depth overview of the latest developments in IgAN management, including traditional supportive care and exposure to novel and specific treatments.

The review highlights the morbidity of IgAN, its risk of progression to ESRD, and the importance of supportive care. New supportive treatments, such as sodium-glucose cotransporter-2 (SGLT2) inhibitors and sparsentan, demonstrate renal protective effects. As for active management, corticosteroids remain effective but are constrained by their side effects. New specific agents, such as budesonide, complement inhibitors, and immune modulators, are showing potential effectiveness in improving proteinuria and preserving renal function. Nonetheless, it faces certain challenges, including a lack of head-to-head trials, limited long-term efficacy and safety data, and affordability issues. The development of IgAN management has considerable potential, although additional research and the implementation of these findings are crucial to optimizing outcomes for patients worldwide.

## Introduction and background

The management of immunoglobulin A nephropathy (IgAN) traditionally has been based on optimized supportive therapy using basic components, including blood pressure management, renin-angiotensin system (RAS) inhibition, dietary and lifestyle interventions, and treatment of cardiovascular risks. However, the effectiveness and safety of broad immunosuppression have been debated. The substantial benefit to most patients has been repeatedly highlighted as the basis for supportive measures. However, indiscriminate adoption of corticosteroids or cytotoxic therapy carries a risk of harm that is unlikely to lead to long-term sustainability of the kidneys [1].

Over the last five years, there has been a shift in the therapeutic approach to IgAN, with an initial focus on supportive therapeutic protocols. In contrast, current therapeutic approaches focus on disease mechanisms. Many complementary developments caused this change. First, large cardiovascular/chronic kidney disease (CKD) outcomes trials and prespecified subgroup analyses have suggested that sodium-glucose cotransporter-2 (SGLT2) inhibitors (SGLT2i, such as dapagliflozin) slow the progression of chronic kidney disease in patients with glomerular diseases, including IgAN, and provide a broadly applicable, well-tolerated nephroprotective class of supportive measures. These results justify the use of SGLT2i in modern patient management algorithms for proteinuric IgAN [2].

Second, potent IgAN-targeted therapies that aim to disrupt specific pathogenic pathways within the gut-kidney axis have progressed through pivotal investigations and attained regulatory approval. Targeted-release budesonide (NEFECON/TARPEYO), a steroid localized to the ileocecal Peyer’s patches, where pathogenic galactose-deficient IgA1 is produced, lowered proteinuria and met regulatory surrogate endpoints. The agent was granted accelerated approval and, subsequently, following additional supportive extension data, became the first therapy approved specifically for IgAN in the United States and Europe [3].

Third, sparsentan, a dual endothelin-angiotensin receptor antagonist, exhibited more pronounced and longer-lasting antiproteinuric responses and positive estimated glomerular filtration rate (eGFR) slope signals compared to irbesartan in the phase 3 PROTECT trial, indicating that hemodynamically and endothelin-modulating non-immunosuppressive strategies can directly alter the course of the disease [4].

Recent research has underscored the growing role of complement inhibitors and immunomodulators in redefining IgA nephropathy (IgAN) treatment. Complement pathway activation - particularly the alternative and lectin pathways - has been identified as a key driver of glomerular injury in IgAN. Novel agents such as iptacopan (LNP023), a selective factor B inhibitor, have shown promising results in phase II trials by significantly reducing proteinuria and preserving kidney function, with phase III studies (APPLAUSE-IgAN) confirming its renoprotective effects [5]. Similarly, narsoplimab, a mannan-binding lectin-associated serine protease-2 (MASP-2) inhibitor targeting the lectin pathway, demonstrated substantial proteinuria reduction and improved eGFR stability [6]. In parallel, immune-modulating biologics targeting B-cell activating factors (BAFFs), such as sibeprenlimab, an anti-APRIL (a proliferation-inducing ligand) monoclonal antibody, have achieved over 40% reductions in proteinuria in phase II studies [7]. These findings suggest that complement blockade and immune modulation offer disease-specific, non-steroidal approaches, representing a transformative shift toward precision immunotherapy in IgAN management.

Following these developments, the recent IgAN guidelines acknowledge a paradigm shift: Kidney Disease: Improving Global Outcomes (KDIGO) now recognizes that treatment goals should not be limited to generalized immunosuppression and instead recommends rigorous supportive care combined with available therapies targeting pathogenic IgA production or the specific intrarenal pathway involved. The modern landscape is a combination of optimized background therapy (blood pressure management, RAS blockade, SGLT2i, and dietary measures) and evidence-based targeted therapy in selected patients. However, further trials currently underway are narrowing the scope of patient selection, timing, and combination.

This review aims to capture current developments in the management of IgAN, including both supportive and novel targeted therapies. It is devoted to evaluating the effectiveness and limitations of existing treatments, as well as emerging pharmacologic advances. New therapeutic agents such as SGLT2i, dual endothelin-angiotensin receptor antagonists, targeted-release budesonide, complement inhibitors, and immunomodulators are discussed, with emphasis on their mechanisms of action, clinical trial outcomes, and impact on renal function and proteinuria. Furthermore, this review assesses how these innovative therapies are reshaping clinical recommendations and long-term disease management strategies for patients with IgAN.

## Review

IgAN

The most prevalent primary glomerulonephritis is IgAN, a disease characterized by the deposition of immunoglobulin A (mainly IgA1) in the glomerular mesangium, resulting in an increase in matrix, inflammation, and eventual damage [8]. Its clinical course is widely variable, ranging from benign hematuria to progressive CKD, with a significant proportion attaining ESRD. In recent years, advances in understanding pathogenesis and emerging clinical trial evidence have begun to shift the treatment paradigm from predominantly nonspecific to a focused approach aimed at altering primary pathogenic pathways [9]. Simultaneously, clinical data and prolonged observational levels of analysis contribute to characterizing prognostic risk stratification and unmet resource gaps in therapy. This happens at an opportune time: with approved agents like sparsentan and targeted-release budesonide now on the scene, the field is finding itself in a place where therapeutic options are designed to match the pathophysiology of the disease, rather than occurring randomly. However, there are some issues, including comparative efficacy, long-term outcomes, and accessibility.

Disease burden and risk of ESRD

Nephropathy at IgA imposes a significant burden on patients, healthcare, and societies, mostly because significant portions of patients might experience a subsequent transition to CKD and ESRD. Long-term cohort research shows that despite optimum supportive measures, ESRD occurs in 20%-40% of patients after 10-20 years, although life expectancy varies based on regional and risk disparities in the timing of onset [10]. In 1,155 adults of the Chinese cohort, 10-, 15-, and 20-year renal survival rates were 83, 74, and 64, respectively; at 36 years of age, one out of every five patients had attained ESRD [11].

Important clinical predictors of adverse outcomes have been confirmed in the general population. The most potent modifiable risk factor is persistent proteinuria (particularly proteinuria above 1 g/day) or time-average proteinuria [12]. Reduced eGFR at presentation, hypertension, elevated mean arterial pressure, male gender, and poor histologic characteristics (Chinese, Oxford classification-like, M, E, S, T, and C lesions) all contribute to an elevated risk of progression. Several risk prediction tools have incorporated clinical and biopsy data (e.g., the IgAN Risk Prediction tool) to risk-select patients who will progress to >50% eGFR loss or ESRD by five years, but none are yet used prospectively [13].

The burden is increased by CKD complications, increased risk of cardiac disease, and the high cost of dialysis or transplantation. ESRD imposes huge health care costs, loss of productivity, poor quality of life, and increased mortality [14]. Moreover, moderate CKD may also start years before ESRD, with its implications of the risk of cardiovascular disease, anemia, bone-mineral issues, and hospital admissions. The disease's natural history complicates the design of clinical trials, necessitating risk stratification and time-scaling interventions early in life [15]. New therapeutic agents are aimed at preventing progression at earlier stages, reducing proteinuria, and conserving renal parenchymal stores before the build-up of deleterious damage.

Pathophysiological insights into IgAN

IgAN has since been conceptualized as a multi-hit mechanism in which the aberrant production of IgA1 results in glomerular injury through the action of a cascade of interrelated immunological events. In recent years, this model has been formalized with added understanding of genetic orientation, complement activation, mucosal immune disruption, and transferable/fibrotic reactions [16].

Patients affected by IgAN-type produce an IgA1 molecule that has abnormal O-glycosylation in the hinge (i.e., galactose-deficient, or Gd-IgA1). Genetic risk loci (e.g., in glycosylation enzymes) and potentially environmental precipitants cause this aberrancy. More recent research is pointing toward cytokines like APRIL and BAFF in the development and survival of B-cells and plasma cells in gut-associated and other mucosal lymphoid tissues that generate Gd-IgA1. The contribution of dysbiosis in the gut microbiome could be impacted by disrupting mucosal immune balance [17].

Exposure of the abnormal hinge area (neo-epitope) of Gd-IgA1 stimulates the development of autoantibodies (IgG or IgA) that identify such defective glycan forms. Gd-IgA1 in circulation is bound by these autoantibodies. High concentrations of these autoantibodies are associated with the disease's aggressive nature [18]. Gd-IgA1 complexed with autoantibodies creates circulating immune complexes (CICs). These CICs have transformed the clearance and physicochemical characteristics (e.g., size, affinity) that prefer mesangial deposition. Solvable variations of IgA receptor (CD89) and mesangial receptors (CD71/transferrin receptor) [19] facilitate the deposition process.

CICs, after deposition into the mesangium, stimulate mesangial cell proliferation, extracellular matrix expansion, release of cytokines and chemokines (e.g., interleukin-6 (IL-6) and tumor necrosis factor-α (TNF-α)), and complement activation (particularly of the alternative and lectin pathways). It causes inflammation of glomeruli, destruction of the glomerular filtration barrier (resulting in proteinuria), and provokes podocyte damage, tubular damage, and interstitial fibrosis. The worst outcomes are associated with the mesangial hypercellularity, endocapillary proliferation, enlarged segmental sclerosis, and interstitial fibrosis/ tubular atrophy histologic features [20].

Complement dysregulation plays a significant amplifying role: the presence of C3, C5b-9, properdin, etc., in glomeruli is associated with an increased risk. Modulators, genetic variants (factor H gene and others), regulate the activity of the complement pathway and have an impact on susceptibility and progression. More and more, environmental triggers (e.g., mucosal infections, microbiome perturbations) are recognized as disease activity modulators [21]. In addition to the glomerular injury, tubulointerstitial destruction is critical in the pathogenesis of the disease. Proteinuria, in itself, is toxic to tubular epithelial cells, and inflammatory mediators, oxidative stress, and ischemia also contribute to the atrophy of tubules. Recent transcriptomic studies propose cell-state shifts by which mesangial cells can become myofibroblast-like, to play a role in matrix deposition and fibrosis. After experiencing fibrosis, it becomes massively irreversible [19].

Role of conservative measures

The standard of conservative or supportive care is still a primary parameter in the treatment of IgAN. The indicated strategies will help to minimize proteinuria, preserve renal function, and reduce progression to ESRD without systemic treatment toxicities. Among the major components are blood pressure management, renin-angiotensin-aldosterone system (RAAS) inhibition, lifestyle and diet therapy, cardiovascular risk factor management, and monitoring [22].

Optimal therapeutic doses of the ACE inhibitors or angiotensin receptor blockers (ARBs) to achieve proteinuria and glomerular pressure minimum are well known. Randomized controlled trials and meta-analyses demonstrated that the RAAS blockade can reduce proteinuria and reduce the rate of eGFR reduction [23]. In practice, the Supportive Versus Immunosuppressive Therapy for the Treatment of Progressive IgA Nephropathy (STOP-IgAN) study involved a run-in period during which further optimization of conservative therapy (including maximal RAAS blockade) was achieved; some participants achieved proteinuria remission during this run-in period and were not randomized to treatment [8]. Protein levels above 1 g/day are associated with a significantly increased risk of adverse kidney outcomes [24].

Supportive care also includes sodium restriction to achieve better antihypertensive and antiproteinuric effects, dietary protein control, lipid control, weight control, smoking cessation, and reduction of other comorbidities (e.g., diabetes, obesity). Research studies show that, even in normotensive IgAN patients, sodium limitation has the potential to lower proteinuria by increasing the inhibitory effect of RAAS to a higher level [25]. The low-protein diets unique to IgAN are less supported by evidence, but they are generally fitting for CKD management [26].

Frequent monitoring of proteinuria, eGFR, blood pressure, and histologic risk characteristics is essential in the management of IgAN. Proteinuria is considered both a clinical outcome and a surrogate endpoint because reductions in proteinuria are strongly associated with slower CKD progression and a lower risk of kidney failure [24].

The KDIGO 2025 Clinical Practice Guideline for IgAN and IgA Vasculitis recommends that optimal supportive care be established before considering immunosuppressive therapy. The primary goals of treatment are to reduce proteinuria, preserve kidney function, delay progression to kidney failure, and minimize treatment-related toxicity through a risk-stratified approach. This includes maximized RAS blockade, strict blood pressure control, lifestyle modification, and the use of SGLT2i when appropriate [27].

According to the Cure Glomerulonephropathy Network (CureGN) study, although around 70% of patients were receiving a renin-angiotensin-aldosterone system inhibitor (RAASi), few (approximately 40%) achieved blood pressure control or complete remission of proteinuria despite treatment [28]. This is a suboptimal utilization of appreciated conservative actions, implying missed opportunities to delay progression.

Optimized supportive therapy alone can lead to clinically meaningful positive outcomes in patients at lower risk (lower proteinuria, higher eGFR, less histologic damage). For instance, during the run-in supportive care-only phase of the STOP-IgAN, patients with adequate protection against proteinuria no longer qualified for the immunosuppressive arm of the trial [24]. But in patients with increased risk, supportive treatment is frequently inadequate, leading to an attempt to try either immunosuppression or new modalities.

Role of corticosteroids: benefits and limitations

Corticosteroids have been used in the treatment of IgAN for many years, particularly in patients with persistent proteinuria who remain at high risk of disease progression despite optimized supportive care. Earlier evidence supporting corticosteroid therapy was provided by the Pozzi trial, which demonstrated significant reductions in proteinuria and improved long-term renal outcomes in patients with IgAN [29]. Their role has become clearer in recent years following the publication of several important studies that have helped define both their benefits and limitations.

The Therapeutic Evaluation of Steroids in IgA Nephropathy Global (TESTING) trial demonstrated that reduced-dose methylprednisolone significantly reduced proteinuria and the risk of major kidney outcomes, including a substantial decline in eGFR, kidney failure, and kidney-related death, compared with supportive care alone [30]. In addition, previous reviews and expert analyses have supported the use of corticosteroids in carefully selected patients while emphasizing the need to balance potential benefits against treatment-related toxicity [31].

Nevertheless, the evidence has not been uniformly positive. The STOP-IgAN trial found that adding immunosuppressive therapy, including corticosteroids, to intensive supportive care did not provide a significant long-term improvement in renal outcomes, despite early reductions in proteinuria, and was associated with a higher rate of adverse events [32]. Other observational studies and meta-analyses, including analyses from the VALIGA cohort, have reported favorable effects of corticosteroid-based regimens on proteinuria and remission rates, although these findings should be interpreted cautiously because of study limitations and heterogeneity among patient populations [33,34,35].

Importantly, corticosteroid therapy remains associated with significant adverse effects, including serious infections, weight gain, glucose intolerance, hypertension, and other metabolic complications, which may limit its use in certain patients [29,32,34].

Overall, corticosteroids continue to have a role in selected patients with IgAN, but their use should be individualized. The potential renal benefits must be carefully weighed against the risk of adverse effects, particularly infections and metabolic complications, and treatment decisions should be guided by the patient's overall risk profile and response to supportive therapy (Table 1).

**Table 1:** Major clinical trials evaluating corticosteroid therapy in IgA nephropathy (IgAN). STOP-IgAN, Supportive Versus Immunosuppressive Therapy for the Treatment of Progressive IgA Nephropathy; TESTING, Therapeutic Evaluation of Steroids in IgA Nephropathy Global; VALIGA, Validation Study of the Oxford Classification of IgA Nephropathy; eGFR, estimated glomerular filtration rate

Trial	Study design/no. of patients	Inclusion criteria	Corticosteroid regimen	Main outcomes	Major adverse events
STOP-IgAN Trial [32]	Multicenter randomized controlled trial; *162 patients*	Biopsy-proven IgAN, persistent proteinuria >0.75 g/day despite six months of optimized supportive care, eGFR >30 mL/min/1.73 m²	Oral corticosteroids alone or combined with cyclophosphamide/azathioprine, depending on GFR	Early reduction in proteinuria, but no significant long-term renal survival benefit compared with supportive care alone	Increased infections, weight gain, impaired glucose tolerance, and metabolic complications
TESTING Trial [30]	International randomized controlled trial; *503 patients*	Biopsy-confirmed IgAN, proteinuria ≥1 g/day despite supportive care, eGFR 20-120 mL/min/1.73 m²	Oral methylprednisolone (initial high-dose protocol later modified to reduced-dose regimen) for six to nine months	Significant reduction in risk of 40% eGFR decline, kidney failure, or renal death; marked reduction in proteinuria	Serious infections, hospitalization, steroid-induced diabetes, gastrointestinal bleeding, and infection-related deaths in the high-dose arm
VALIGA Study [35]	European multicenter observational cohort; *1,147 patients*	Biopsy-proven IgAN with variable proteinuria and preserved kidney function	Systemic corticosteroid therapy in selected high-risk patients	Corticosteroid-treated patients showed slower renal function decline and lower proteinuria	Higher frequency of steroid-related adverse metabolic effects and infections
Pozzi et al. Trial [29]	Randomized controlled trial; *86 patients*	IgAN with proteinuria 1-3.5 g/day and preserved renal function	Intravenous methylprednisolone pulses at months 1, 3, and 5 plus oral prednisone on alternate days for six months	Improved renal survival and lower proteinuria over long-term follow-up	Weight gain, hypertension, glucose intolerance, and steroid-related adverse effects

Emerging role and advances in supportive measures

The supportive therapy in IgAN over the past few years, however, has evolved with the introduction of new agents that not only maintain blood pressure but also decrease proteinuria through the inhibition of RAAS. SGLT2i and sparsentan (a dual endothelin type A receptor antagonist + angiotensin II receptor blocker) are among the most promising ones, and both are demonstrating significant reductions in proteinuria and retarded progression of kidney function.

SGLT2i

According to the DAPA-CKD trial, 270 patients qualified, with biopsy-proven or clinically confirmed IgAN, a recent combination stalemate of dapagliflozin (10 mg g/day) together with conventional therapy (this went on either to ACE or ARB) yielded statistically significant benefits as a 50% or greater reduction in the risk of renal failure or eGFR, or the elimination of the causes of conventional therapy. The hazard ratio (HR) was approximately 0.29 (95% confidence interval (CI) 0.12-0.73), indicating approximately 71% reduction in relative risks (composite endpoint). The dapagliflozin arm exhibited a slower rate of eGFR decline (3.5 mL/min/1.73 m²/year) compared with the placebo arm (4.7 mL/min/1.73 m²/year). In addition, the urinary albumin-to-creatinine ratio (UACR) decreased by approximately 26% [2].

The EMPA-KIDNEY trial (Empagliflozin in Patients with Chronic Kidney Disease) further reinforced the renoprotective role of SGLT2i in IgAN. This large, randomized, double-blind, placebo-controlled study evaluated empagliflozin (10 mg daily) versus placebo in more than 6,600 participants with CKD, including a prespecified subgroup of 817 patients with biopsy-confirmed IgAN [36]. In this subgroup, empagliflozin significantly reduced the risk of kidney disease progression or cardiovascular death by approximately 28% compared with placebo, consistent with the overall population results. Importantly, the benefit was independent of diabetes status and baseline eGFR, confirming that SGLT2i provide robust kidney protection even in non-diabetic glomerular diseases. Proteinuria was also reduced by roughly 25%-30% within six months of therapy, and safety outcomes were favorable, with no excess of serious adverse events compared with placebo. These results established empagliflozin as an evidence-based therapeutic option for patients with IgAN, supporting its inclusion in treatment guidelines as part of comprehensive supportive care.

Real-world studies support these advantages. As an example, a prospective comparative study (n = 44) of RAAS inhibitors and subsequent corticosteroid therapy in Japanese patients revealed that dapagliflozin produced the most significant reduction in proteinuria in patients with a baseline urine protein-to-creatinine ratio (UPCR) exceeding 0.5 g/g and mitigated the post-treatment decline in eGFR, particularly in patients with higher baseline proteinuria levels [37].

One more (also real-world) study in more than 60 patients with IgAN observed approximately 25%-30% improvements in proteinuria over six months of SGLT2i-based therapy. The difference was that an early decrease in eGFR (temporal effect) at the time of starting treatment was a predictor of higher reductions in proteinuria at 12 months of follow-up [38].

Sparsentan

Sparsentan (FILSPARI 400 mg/day) has been evaluated in the PROTECT trial, a phase 3 randomized, double-blind, active-controlled, global study comparing sparsentan (400 mg/day) with irbesartan (300 mg/day) in 404 patients with IgAN who had persistent proteinuria despite maximal RAAS inhibition. At week 36, sparsentan demonstrated a significantly greater reduction in baseline proteinuria compared with irbesartan, with a mean reduction of 49.8% versus 15.1% in UPCR (g/g), thereby meeting the primary efficacy endpoint. These findings highlighted sparsentan’s dual endothelin and angiotensin receptor blockade as an effective, non-immunosuppressive therapeutic option capable of substantially reducing proteinuria and improving renal outcomes in patients at high risk of progression. More sustained benefits were observed in the longer-term (110-week) analysis, with sparsentan achieving greater reductions in proteinuria and a slower rate of eGFR decline compared with irbesartan. The difference in chronic eGFR slope favored sparsentan and reached statistical significance (*P* = 0.037), supporting its potential long-term renoprotective effects in patients with IgAN [39].

An exercise of sparsentan therapy in combination with RAS + SGLT2i background with a real-world managed access program (23 patients) yielded proteinuria reductions (which was faster and clinically meaningful) at very high levels (up to ~62% reduction at 14 weeks) in combination with the available standard of care treatments, again indexing the additive efficacy of specific standard of care treatments [40].

Emerging advances (novel and targeted therapies)

Targeted-Release Budesonide

Targeted-release budesonide (Nefecon/Tarpeyo) integrates the corticosteroid directly into the ileocecal Peyer’s patches (a proposed site of mucosal pathogenic galactose-deficient IgA1 production). It therefore aims to inhibit the activation of B-cells in the system through topical deposition while reducing doses of systemic steroid use. Proteinuria and a favorable eGFR slope response to systemic high-dose steroids were demonstrated to be marked and long-lasting, lower than placebo, with an excellent safety profile. A nine-month NefIgArd trial evaluated targeted-release budesonide (16 mg once daily) in 199 adult patients with biopsy-proven IgAN, persistent proteinuria (≥1 g/day), and eGFR ≥35 mL/min/1.73 m² despite optimized supportive therapy, including RAS blockade. The study demonstrated significant reductions in proteinuria compared with placebo and a slower decline in renal function over the treatment period. Still, long-term hard outcomes (ESRD) and other adverse events are not well quantified due to the absence of sustained surveillance. The mechanism of action and oral route, combined with approval by major jurisdictions for targeted budesonide, make it broadly available as an IgAN-specific therapy. It serves as an effective steroid-sparing option, particularly in cases where there is a high risk of systemic corticosteroid toxicity [41].

Complement Inhibitors

Complement activation plays a pivotal role in the pathogenesis of IgAN, particularly through the alternative and lectin pathways, which contribute to glomerular inflammation and mesangial injury. Recent advances have led to the development of several complement-targeting agents showing promise in clinical trials. Iptacopan (LNP023), an oral factor B inhibitor that selectively blocks the alternative pathway, demonstrated significant proteinuria reduction and stabilization of eGFR in phase II trials, with phase III results from the APPLAUSE-IgAN study (2024) confirming its renoprotective efficacy and favorable safety profile [5]. Similarly, narsoplimab, a monoclonal antibody against MASP-2, inhibits the lectin pathway and has been associated with marked reductions in proteinuria and improved renal function in phase II studies [6]. Other complement inhibitors, including ravulizumab (C5 inhibitor) and cemdisiran (C5 mRNA silencer), are currently under clinical investigation for IgAN, with no established routine clinical use yet. At present, complement-targeted therapy is being explored mainly in patients with progressive IgAN characterized by persistent proteinuria despite optimized supportive care (including RAAS blockade and SGLT2i), particularly in those with evidence of active complement activation on kidney biopsy (e.g., C3/C5b-9 deposition) or a high-risk profile for rapid eGFR decline. These agents are therefore not yet recommended in guidelines for standard care but are being evaluated as potential precision therapies for selected high-risk subgroups in clinical trials, where they may offer targeted modulation of complement activity while avoiding broad systemic immunosuppression.

Immune Modulators

Novel immunomodulatory strategies in IgAN are increasingly focused on the B-cell and plasma-cell axis, aiming to reduce the production of pathogenic galactose-deficient IgA1 and its corresponding autoantibodies. Agents targeting the APRIL and BLyS (B-lymphocyte stimulator) pathways, such as atacicept, telitacicept, and povetacicept, have shown promising results in phase II clinical trials, achieving sustained and clinically meaningful reductions in proteinuria with an acceptable safety profile and manageable adverse effects [9]. These therapies work by suppressing the survival and differentiation of plasma cells that secrete aberrant IgA, thereby directly targeting the immunopathogenic mechanism of the disease. In contrast, conventional anti-CD20 therapy (rituximab), which depletes circulating B cells, has shown limited efficacy in randomized controlled trials-likely because long-lived plasma cells, which are CD20-negative, continue to produce pathogenic IgA antibodies. Consequently, future IgAN management will likely depend on therapies that selectively target plasma cells, BAFF/APRIL signaling, or combinations thereof, offering a more precise and durable disease-modifying approach. Ongoing phase III trials and biomarker-based sub-studies are expected to further define their long-term efficacy, safety, and infection risk profiles.

Felzartamab, a fully human anti-CD38 monoclonal antibody, is another emerging immunomodulatory therapy under investigation for IgAN. Targeting CD38-positive plasma cells involved in the production of pathogenic galactose-deficient IgA1 and related autoantibodies, it may offer a novel disease-modifying approach. In the randomized, double-blind, placebo-controlled phase 2a IGNAZ trial, felzartamab was associated with significant reductions in proteinuria and stabilization of kidney function, with an acceptable safety profile [42]. However, these findings are based on a relatively small phase 2 study, and larger studies with longer follow-up are needed to establish its long-term efficacy and safety.

Overall, the treatment landscape of IgAN has evolved substantially, with multiple targeted therapies demonstrating favorable efficacy and safety outcomes in recent comparative analyses [43].

**Figure 1:**
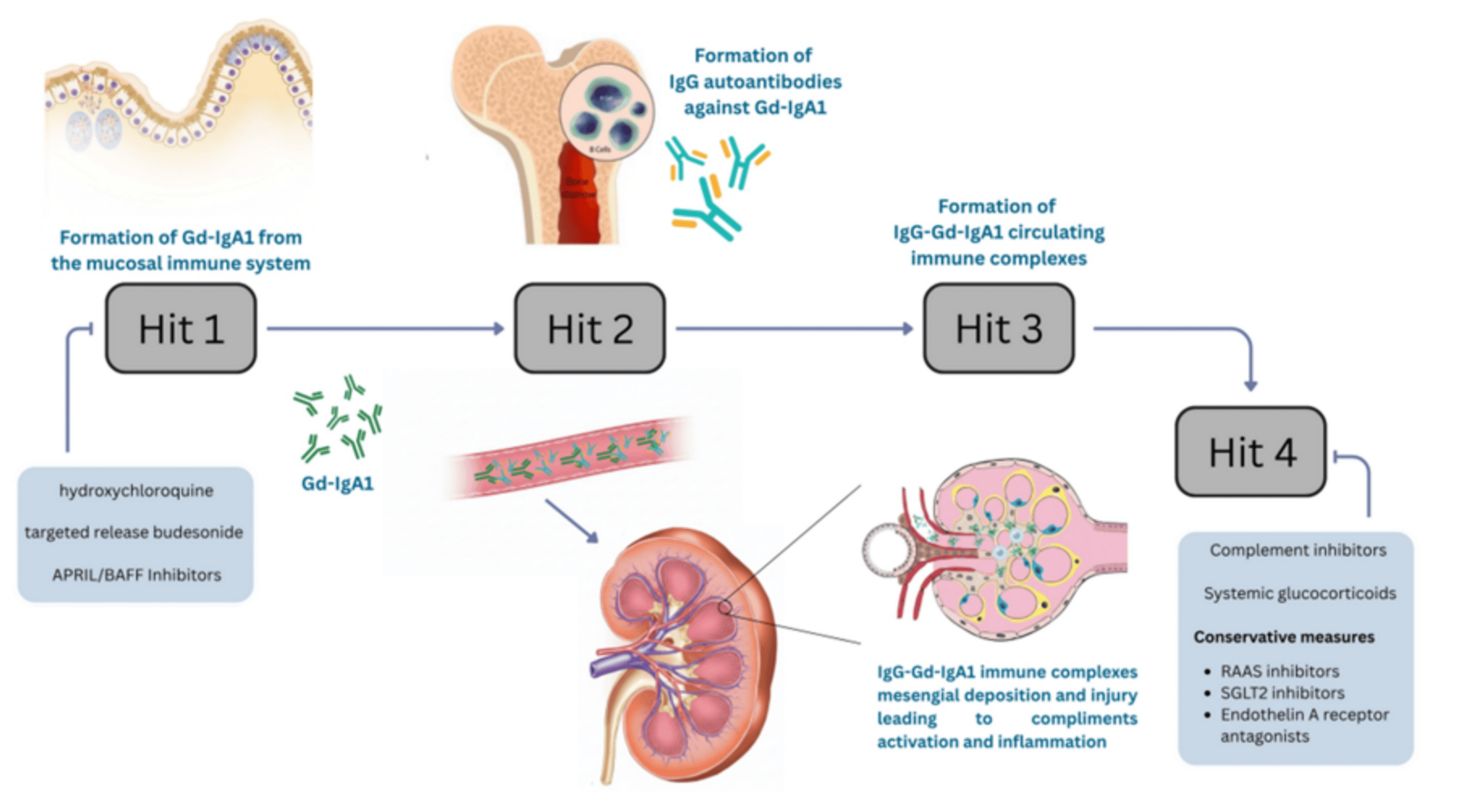
Pathogenic mechanisms and emerging immunomodulatory strategies in IgA nephropathy (IgAN). This figure was self-created by BioRender (BioRender Inc., Toronto, ON, Canada) and Adobe Illustrator (Adobe Inc., San Jose, CA). IgA, immunoglobulin A; RAAS, renin-angiotensin-aldosterone system; SGLT2, sodium-glucose cotransporter-2; APRIL, a proliferation-inducing ligand; BAFF, B-cell activating factor

Figure 1 illustrates pathogenic mechanisms and different treatment strategies in IgAN.

Unmet needs and future challenges

Lack of Head-to-Head Comparative Trials

A persistent limitation in IgAN research is the absence of direct head-to-head randomized controlled trials comparing emerging therapies. Although network meta-analyses suggest that multiple drug classes-including APRIL/BLyS pathway inhibitors, complement factor B antagonists, sparsentan, and targeted-release budesonide-are effective compared with supportive care, there is still no robust evidence establishing their relative superiority against each other. For example, the Proteinuria Reduction with Sparsentan in IgA Nephropathy (PROTECT) trial evaluated sparsentan versus irbesartan, whereas budesonide trials primarily compared treatment with placebo; however, no studies have directly compared sparsentan with budesonide or complement inhibitors, nor have they evaluated combination regimens such as sparsentan plus budesonide or complement blockade [39,44].

Absence of Long-Term Efficacy and Safety Data

Although newer therapies such as targeted-release budesonide and sparsentan demonstrate promising short- to medium-term outcomes, long-term efficacy and safety remain insufficiently established. The BUDIGAN open-label extension study reported sustained reductions in proteinuria and preservation of eGFR over 36 months with budesonide, with generally mild adverse effects [45]. Similarly, interim results from the PROTECT trial suggest sustained proteinuria reduction and favorable eGFR slope over approximately 36 weeks; however, hard renal outcomes such as end-stage kidney disease (ESKD), dialysis initiation, or mortality remain unconfirmed [39]. Given that IgAN typically progresses over 10-20 years, current follow-up durations are inadequate to fully assess disease-modifying potential.

Cost and Accessibility of Novel Therapies

Despite clinical advances, new IgAN therapies remain costly and are not uniformly accessible across different healthcare systems. For instance, cost-effectiveness analyses of targeted-release budesonide in the United States estimate incremental costs of approximately $3,810 compared with supportive care, with an incremental cost-effectiveness ratio (ICER) of about $17,538 per quality-adjusted life year (QALY). While this may be acceptable in high-income healthcare settings, it remains prohibitive in low- and middle-income countries. The KDIGO guidelines emphasize that regulatory approval does not necessarily ensure affordability or equitable access, particularly in resource-limited settings [46]. Consequently, disparities in access to advanced therapies may widen global treatment inequities in IgAN management.

Emerging Role of Combination and RNA-Based Therapies

Future therapeutic strategies are increasingly focusing on combination approaches and novel molecular platforms. There is growing interest in combining agents that target different pathogenic pathways, such as gut mucosal immunity modulation (targeted-release budesonide), endothelin-angiotensin receptor blockade (sparsentan), and complement inhibition, to achieve additive or synergistic effects on proteinuria reduction and disease progression. However, these combinations have not yet been evaluated in large randomized clinical trials.

RNA-targeting therapies are also emerging as a potential future direction in IgAN treatment. Examples include IONIS-FB-LRx, an antisense oligonucleotide targeting complement factor B of the alternative pathway, and ARO-C3, an RNA interference therapy designed to reduce complement C3 production. Although these agents remain in early clinical development, they highlight the growing shift toward precision-based and pathway-specific therapies in IgAN [47].

## Conclusions

IgAN has moved past the era of traditional supportive care to the era of focused therapies. New approaches, including SGLT2, sparsentan, budesonide, immunomodulators, and complement inhibitors, have proven significant proteinuria reduction and stabilized renal performance, which provides hope of improvement in comparison to the classic renin-angiotensin system blockade and systemic steroids. Despite recent advances in IgAN treatment, key challenges remain, including the lack of head-to-head clinical trials, limited long-term safety data, and issues of cost and accessibility. These challenges are further complicated by significant disease heterogeneity across different populations, which affects how patients present and respond to therapy. In addition, there is still a need for better early identification of high-risk patients and standardized diagnostic and classification systems. Without these, implementing consistent, tiered treatment strategies remains difficult. Future approaches should therefore focus not only on comparative and long-term clinical evidence but also on improving diagnosis, risk stratification, and globally standardized treatment frameworks.
